# Syphilis in HIV-infected patients: predictors for serological failure and serofast state

**DOI:** 10.7448/IAS.15.6.18106

**Published:** 2012-11-11

**Authors:** R Palacios, F Navarro, T Moreno, J Ruiz, E Nuño, M Márquez, J Santos

**Affiliations:** Hospital Virgen de la Victoria, Málaga, Spain

## Abstract

**Purpose:**

HIV-infected patients treated for syphilis may be at increased risk for serological failure and serofast state. Our aim was to analyse serological response to treatment in HIV-infected patients diagnosed with syphilis, and factors associated with serological cure and serofast state.

**Methods:**

Open-label, no controlled study of a series of HIV-patients diagnosed with syphilis during 2004–2011. Patients were categorized by rapid plasma reagin titer (RPR) into success (4-fold decrease in RPR by 12 or 24 months after treatment of early or late syphilis), serofast (success with persistently stable reactive RPR), and failure/re-infection (failure to decrease 4-fold in RPR by 12 or 24 months after treatment or sustained 4-fold increase in RPR after treatment response).

**Results:**

141 HIV-patients were diagnosed with syphilis during the study period (104 early syphilis, 36 late or indeterminate latent syphilis). The mean age was 36.3 years, 98.5% were male, and 87.2% homosexual men. In 46 (32.6%) cases, HIV and syphilis infection diagnosis were coincident (mean CD4 457/mm^3^ and HIV-VL 4.72 log_10_). Among patients with prior known HIV infection, 65 were on antiretroviral therapy (ART) at syphilis diagnosis (mean CD4 469/mm^3^, 76.9% undetectable HIV-VL). 116 patients satisfied criteria for serological response analysis (89 early, 24 late/indeterminate). At 12 months of early syphilis treatment (89.2% penicillin) there were 16 (18%) failures, and at 24 months of late/indeterminate syphilis (91.7% penicillin) there were 5 (18.5%) failures. Overall, 36 (31.0%) patients presented serofast state. Treatment failure was related with lower CD4 count (295 *vs* 510/mL; p=0.045) only in patients with coincident diagnosis. Serofast state was related with older age (41 *vs* 36 years; p=0.024), and lower CD4 count (391 *vs* 513/mm^3^; p=0.026).

**Conclusions:**

In this series of HIV-infected patients, with many patients on ART and with good immunological and virological parameters, serological failure and serofast state were frequent. Immunological status, and age could influence on serological response to syphilis treatment in HIV-infected patients.

